# α‐Mangostin suppresses the de novo lipogenesis and enhances the chemotherapeutic response to gemcitabine in gallbladder carcinoma cells via targeting the AMPK/SREBP1 cascades

**DOI:** 10.1111/jcmm.14785

**Published:** 2019-11-25

**Authors:** Yu Shi, Yangwei Fan, Yuan Hu, Jiayu Jing, Chuying Wang, Yinying Wu, Qianqian Geng, Xuyuan Dong, Enxiao Li, Danfeng Dong

**Affiliations:** ^1^ Department of Medical Oncology First Affiliated Hospital of Xi'an Jiaotong University Xi'an China; ^2^ Department of Gastroenterology Second Affiliated Hospital of Xi'an Jiaotong University Xi'an China; ^3^ Department of Nuclear Medicine First Affiliated Hospital of Xi'an Jiaotong University Xi'an China

**Keywords:** AMPK, gallbladder cancer, gemcitabine, lipogenesis, SREBP1, α‐mangostin

## Abstract

High rates of de novo lipid synthesis have been discovered in certain kinds of tumours, including gallbladder cancer (GBC). Unlike several other tumours, GBC is highly insensitive to standard adjuvant therapy, which makes its treatment even more challenging. Although several potential targets and signalling pathways underlying GBC chemoresistance have been revealed, the precise mechanisms are still elusive. In this study, we found that α‐Mangostin, as a dietary xanthone, repressed the proliferation and clone formation ability, induced cell cycle arrest and the apoptosis, and suppressed de novo lipogenesis of gallbladder cancer cells. The underlying mechanisms might involve the activation of AMPK and, therefore, the suppression of SREBP1 nuclear translocation to blunt de novo lipogenesis. Furthermore, SREBP1 silencing by siRNA or α‐mangostin enhanced the sensitivity of gemcitabine in gallbladder cancer cells. In vivo studies also displayed that MA or gemcitabine administration to nude mice harbouring NOZ tumours can reduce tumour growth, and moreover, MA administration can significantly potentiate gemcitabine‐induced inhibition of tumour growth. Corroborating in vitro findings, tumours from mice treated with MA or gemcitabine alone showed decreased levels of proliferation with reduced Ki‐67 expression and elevated apoptosis confirmed by TUNEL staining, furthermore, the proliferation inhibition and apoptosis up‐regulation were obviously observed in MA combined with gemcitabine treatment group. Therefore, inhibiting de novo lipogenesis via targeting the AMPK/SREBP1 signalling by MA might provide insights into a potential strategy for sensitizing GBC cells to chemotherapy.

## 1 INTRODUCTION

Gallbladder cancer (GBC), the most common malignant biliary tumour, has the seventh highest mortality rate of all gastrointestinal cancers.^1^ Although the incidence of gallbladder cancer is very low at approximately 2.5 cases in 1 × 10^5^ people, GBC prognosis is very dismal with a 5‐year survival rate of 5%.^2^ Currently, gemcitabine/cisplatin is the recognized reference regimen for the first‐line treatment of patients suffering from advanced biliary tract cancers, including gallbladder cancer.^3^ However, patients undergoing the first‐line chemotherapy often have a rapidly worsening performance status, and only a small number of patients are suitable for subsequent treatment.^3^ Unlike other tumours, GBC is especially resistant to the currently available routine adjuvant therapy, thus making GBC management challenging.^4^, ^5^ A few potential targets and signalling pathways underlying GBC chemoresistance have been identified; however, the precise mechanism requires further investigation.^6^, ^7^, ^8^ Thus, studies on the identification of agents that enhance the response to chemotherapeutic drugs and further elucidate the molecular basis of drug resistance are urgent and significant.

Metabolic reprogramming plays an essential role in tumourigenesis and malignant aggravation of cancer.^9^ Elevated aerobic glycolysis and fatty acid synthesis are the most significant aspects of cancer cell metabolism. Newly synthesized lipids are utilized to form the cell membrane during cell proliferation and to supply energy for tumour development. Thus, high rates of de novo lipid synthesis have been detected in numerous types of tumour cells, such as hepatocellular carcinoma,^10^ breast cancer,^11^ pancreatic cancer,^12^ gallbladder cancer, etc.^13^ Sterol regulatory element‐binding proteins (SREBPs) belong to an important family of transcription factors that control gene expression of core enzymes of lipogenesis.^14^ Targeting the key enzymes of lipogenesis is an effective strategy to blunt tumour growth and impair tumour survival.^15^ Notably, previous studies have demonstrated that targeting SREBP1 can abolish the cancer stemness and enhance the chemotherapeutic response to gemcitabine in pancreatic cancer cells.^16^ 5′‐AMP‐activated protein kinase (AMPK), a kinase directly targeting SREBP1, can stimulate phosphorylation at Ser372, inhibit SREBP‐1c cleavage and intranuclear translocation, and suppress the expression of SREBP‐1c target genes in hepatocytes exposed to high levels of glucose, thus decreasing lipogenesis.^17^ Identification of agents that repress lipogenesis might help to provide insights into a promising strategy to enhance the treatment outcome of gallbladder cancer.

As a dietary xanthone, α‐mangostin is mainly isolated from the pericarp of mangosteen or *Garcinia mangostana* L. Previous studies have demonstrated that α‐mangostin has a number of biological activities, including antibacterial,^18^ cardioprotective,^19^ neuroprotective^20^ and anticancer effects.^21^, ^22^ In addition, α‐mangostin kills cancer cells by inducing cell cycle arrest, apoptosis and autophagic cell death; moreover, α‐mangostin suppresses oxidation, invasion and metastasis of several types of cancer.^21^ However, the molecular mechanisms of the effects of α‐mangostin in GBC cells have not yet been reported. Interestingly, α‐mangostin triggers the autophagy‐related cell death of glioblastoma cells by activating AMPK (AMP‐activated protein kinase).^23^ AMPK is a canonical upstream regulator of SREBP1, which is the key transcriptional factor regulating de novo lipid synthesis.

Thus, in this study, we hypothesized that α‐mangostin represses de novo lipogenesis and enhances the chemotherapeutic response to gemcitabine in gallbladder carcinoma cells by targeting the AMPK/SREBP1 cascades.

## 2 MATERIALS AND METHODS

The present study was authorized by the Ethical Committee of the First Affiliated Hospital of Medical College, Xi'an Jiaotong University, China.

### 2.1 Cell culture and reagents

The GBC cell lines, GBC‐SD, and normal biliary epithelial cell line, HIBEC, were acquired from the Shanghai Institute for Biological Science, Chinese Academy of Science (Shanghai, China). NOZ cells were purchased from the Health Science Research Resources Bank (Osaka, Japan). RPMI‐1640 medium containing 10% dialysed foetal bovine serum (FBS) (HyClone), 100 μg/mL streptomycin and 100 U/mL penicillin was used for all cell culture, and the cells were maintained in a humidified atmosphere of 5% CO_2_ at 37°C. MA (C15:1; C22H34O3; molecular weight: 346.50) was purchased from MCE. Gemcitabine was obtained from Selleck Chemicals. Gemcitabine and MA were dissolved in DMSO at the stock concentrations of 10 and 5 mmol/L, respectively. MTT (3‐(4,5‐dimethyl‐2‐thiazolyl)‐2,5‐diphenyl‐2‐H‐tetrazolium bromide) and dye of Oil Red O were purchased from Sigma. Working dilutions of gemcitabine and MA were prepared in the culture medium immediately prior to use, and DMSO was used as the vehicle control. Antibodies targeting SREBP1 and β‐actin were purchased from Santa Cruz Biotechnology; antibodies against FASN (fatty acid synthase), ACC (acetyl‐CoA carboxylase), PCNA (proliferating cell nuclear antigen), Bax, Bcl2, AMPK (AMP‐activated protein kinase), p‐AMPK and Ki‐67 were purchased from Abcam.

### 2.2 Cell viability assay

After seeding the HIBEC, GBC‐SD and NOZ cells into 96‐well plates at a density of 5 × 10^3^ cells per well, a series of concentrations (0, 1, 2, 4, 6, 8, 12 and 16 μmol/L) of MA or various concentrations (0, 10^−3^, 10^−2^, 10^−1^, 10^0^, 10^1^, 10^2^ and 10^3^ μmol/L) of gemcitabine were added. After being transfected with si‐SREBP1 to knockdown SREBP1 for 48 hours, the cells were seeded in 96‐well plates at a density of 5 × 10^3^ cells per well and supplemented with 10 μmol/L gemcitabine for 72 hours. Then, the cell viability was determined by the MTT assay at various time‐points (24, 48 and 72 hours); the absorbance was read with at 490 nm with a multiwell microplate reader (BIO‐TEC Inc).

### 2.3 Colony formation assay

After plating the GBC‐SD and NOZ cells at 1000 cells/well into 35 mm petri dishes and allowing the cells to attach overnight, MA (5 μmol/L) or gemcitabine (10 μmol/L) was used to treat the cells for 24 hours, and the culture medium was replaced with the fresh medium. After 2 weeks of culture, cell colonies were formed. At expected time‐points, the colonies were fixed with 4% paraformaldehyde, stained with 0.1% crystal violet solution, rinsed and imaged; the number of the colonies was counted and statistically evaluated.

### 2.4 Ethynyl deoxyuridine (EdU) incorporation assay

The EdU incorporation assay was conducted using an EdU kit (Roche) according to the manufacturer's instructions. The results were visualized by a Zeiss confocal microscope at a magnification of 200×, and the signals were counted in at least five random fields.

### 2.5 Flow cytometry analysis

We performed flow cytometry analysis to evaluate cell apoptosis and cell cycle progression. We used an Annexin V‐FITC/7‐AAD apoptosis detection kit from Becton Dickinson (BD) to assess apoptosis following the manufacturer's guidelines. In brief, NOZ and GBC‐SD cells were plated into 6‐well plates at a density of 1 × 10^5^ cells/well. After 2 days of treatment, the cancer cells were treated with trypsin, washed with PBS (phosphate buffered saline), stained with Annexin V and 7‐AAD and subjected to flow cytometry analysis. For cell cycle detection, PI/RNase staining buffer (#550825, Becton Dickinson Bioscience) was used to examine the cell cycle progression according to the manufacturer's protocols.

### 2.6 Oil Red O staining

We performed Oil Red O staining to visualize the lipid droplets in the GBC cells. After finishing the designated treatment, the cells were rinsed with PBS three times and fixed in 4% paraformaldehyde for 1 hour. Then, propylene glycol was used to rinse the cells; the cells were stained with pre‐warmed 0.25% Oil Red O working solution in a 60°C oven for 30 minutes. After PBS rinsing, the cell nuclei were visualized by haematoxylin staining. Finally, we photographed the cells using a light microscope (Nikon Eclipse Ti‐S) at 400× magnification.

### 2.7 Western blotting analysis

RIPA lysis buffer was used to extract the total protein; the concentration of the total protein was measured using a BCA protein assay kit (Pierce) following the manufacturer's instructions. The WB assay was conducted as previously reported.^22^ The expression of designated proteins was visualized by enhanced chemiluminescence (Millipore). Images were captured using a ChemiDoc XRS imaging system (Bio‐Rad), and the ImageJ software was used for densitometry analysis of each band. β‐Actin was used as an internal loading control.

### 2.8 Immunofluorescence staining

With implementing the designated treatment, gallbladder cancer cells were fixed in 4% formaldehyde for 30 min, permeabilized with 0.3% Triton X‐100, incubated with 5% BSA (blocking buffer) for 1 hour at room temperature and incubated overnight with a primary antibody at 4°C. The cells were then incubated with a red dye‐conjugated secondary antibody from Jackson ImmunoResearch Laboratories for 1 hour at room temperature; then, the cells were stained with DAPI to visualize the nuclei. Finally, the slides were mounted and observed under a Zeiss confocal microscope at a magnification of 400×.

### 2.9 RNA interference

Loss‐of‐function analysis was performed using siRNAs against AMPK and SREBP1. The sequences of siRNA for AMPK (sense: UUCUCCGAACGUGUCACGUTT; antisense: ACGUGACACGUUCGGAGAATT) and a negative control siRNA (sense: UUCUCCGAACGUGUCACGUTT; antisense: ACGUGACACGUUCGGAGAATT) were designed, and siRNAs were synthesized by GenePharma Co., Ltd. The siRNA sequences for silencing SREBP1 were described previously.^16^ The transfection was conducted as previously reported.^24^ After 24 hour of transfection, the cells were utilized for further experiments.

### 2.10 In vivo tumourigenesis assay

For the subcutaneous tumour formation assay, 1 × 10^6^ of NOZ cells in 100 μL PBS were subcutaneously injected into the left flanks of the 4‐week‐old male BALB/c nude mice (obtained by and housed in the Animal Center at Medical College, Xi'an Jiaotong University). One week after the implantation, the nude mice were randomly divided into the following four groups (6 mice per each group): (a) the control group (administered with sterile water); (b) the MA treatment group (α‐mangostin in a clear solution containing 25% polyethylene glycol (PEG) (2 mg/kg bodyweight) was intraperitoneally injected in a volume of 0.2 mL^23^ daily for 4 weeks); (c) the gemcitabine treatment group (Gem, 40 mg/kg gemcitabine injection via the tail vein, once per week for 4 weeks); and (d) the gemcitabine plus MA group (α‐mangostin in a clear solution containing 25% polyethylene glycol (PEG) (2 mg/kg bodyweight) in a volume of 0.2 mL was intraperitoneally injected daily for 4 weeks in combination with gemcitabine (40 mg/kg) injections once per week for 4 weeks). Tumour growth was continuously monitored by calculating the tumour volume according to the formula: V (tumour volume) = 0.5 × s (shorter diameter)^2^ × L (longer diameter). At the end of the treatment, the nude mice were  sacrificed ; the tumour samples were weighted, fixed and histologically analysed by immunohistochemical staining according to the previously reported procedures.^24^

### 2.11 Statistical analysis

Every experiment was repeated at least three times. The results are shown as the means ± standard deviation. Student's *t* test was performed to compare two groups. One‐way ANOVA followed by the LSD post hoc test was used for statistical analysis of multiple comparison using SPSS (SPSS 18.0; SPSS Inc). *P* < .05 was considered to be statistically significant.

## 3 RESULTS

### 3.1 α‐Mangostin suppresses proliferation and induces apoptosis and cell cycle arrest in gallbladder cancer cells

**Figure 1:**
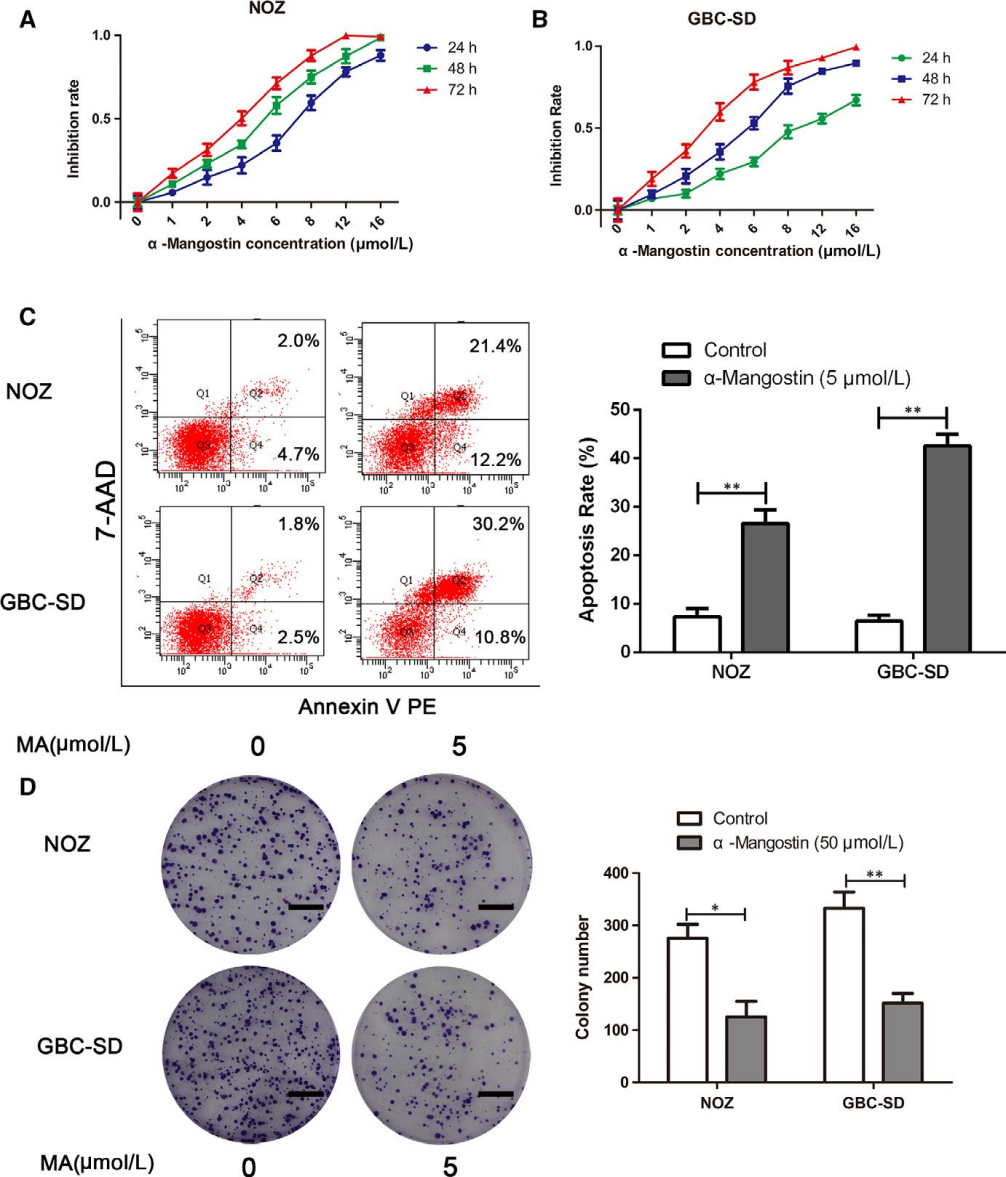
MA treatment supresses the viability and induces apoptosis in gallbladder cancer cells. A, B, Gallbladder cancer cells (GBC‐SD and NOZ) were incubated with gradually increasing concentrations (0, 1, 2, 4, 6, 8, 12 and 16 μmol/L) of MA for 24, 48 and 72 h; the MTT assay was used to assess cell viability. C, GBC‐SD and NOZ cells were treated with MA (5 μmol/L) for 48 h; apoptosis of gallbladder cancer cells was evaluated by flow cytometry. D, Effects of MA on colony formation of GBC‐SD and NOZ cells were detected by colony formation assay. The images are representative of three independent experiments; the colony numbers were counted and plotted. Scale bar, 1 cm. *P < .05, **P < .01. MA, α‐mangostin

**Figure 2:**
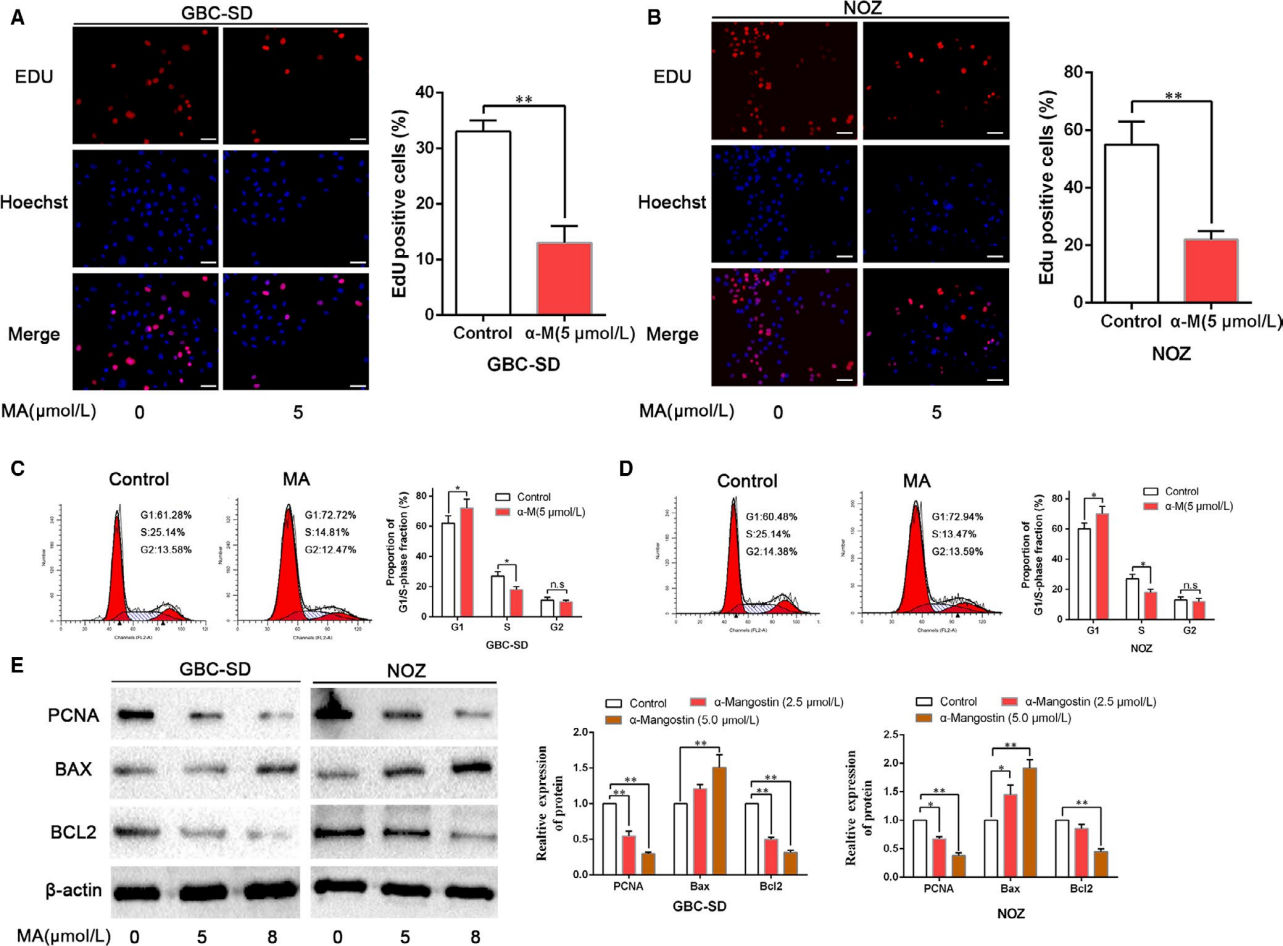
MA impedes proliferation and induces cell cycle arrest in gallbladder cancer cells. A, B, GBC‐SD and NOZ cells were treated with MA, and proliferation was assessed by the EdU assay. Scale bar: 50 μm. C, D, The effects of MA on cell cycle progression of GBC‐SD and NOZ cells were detected by flow cytometry. E, The effects of MA on the expression of proliferation‐ or apoptosis‐related proteins (PCNA, BAX and BCL2) were examined by WB analysis. *P < .05, **P < .01. MA, α‐Mangostin

To evaluate whether MA affects the viability of GBC cells, NOZ and GBC‐SD cells were incubated with a series of incremental doses of MA (0, 1, 2, 4, 6, 8, 12 and 16 μmol/L) to determine whether the effects are time‐dependent. The cell viability was analysed by the assessment of the absorbance at 490 nm after 24, 48 and 72 hours using the MTT assay. As shown in Figure 1A,B, MA repressed the growth of GBC cells in a time‑ and dose‑dependent manner. The half‐maximum inhibitory concentrations of MA were approximately 5 μmol/L in the NOZ and GBC‐SD cells, which were similar to previous results and did not have cytotoxic effects in normal bile duct epithelial cell lines (HIBEC, as shown in Figure S1)^21^; thus, we selected 5 μmol/L MA for the subsequent experiments. To interrogate the effects of MA on the proliferation of GBC cells, NOZ and GBC‐SD cells were treated with 5 μmol/L MA to evaluate colony formation. As displayed in Figure 1D, treatment with 5 μmol/L MA significantly suppressed the number of colonies compared with that in the untreated control group. In addition, the EdU assay revealed that treatment with MA significantly decreased the proliferation of NOZ and GBC‐SD cells (Figure 2A,B). The data from the flow cytometry analysis of NOZ and GBC‐SD cells treated with or without MA (5 μmol/L) for 48 hour are shown in Figure 1C. Treatment of GBC cells with MA led to an increase in the apoptotic population and induced cell cycle arrest compared with those in the untreated control cells (Figure 2C,D). Moreover, the results of Western blot showed that MA treatment increased the expression levels of BAX and decreased the expression levels of PCNA and BCL2 (Figure 2E). Collectively, the data illustrate that MA strongly suppresses the proliferation and induces apoptosis and cell cycle arrest in the GBC cells in vitro.

### 3.2 Inhibition of de novo lipogenesis is achieved with α‐MA treatment

**Figure 3:**
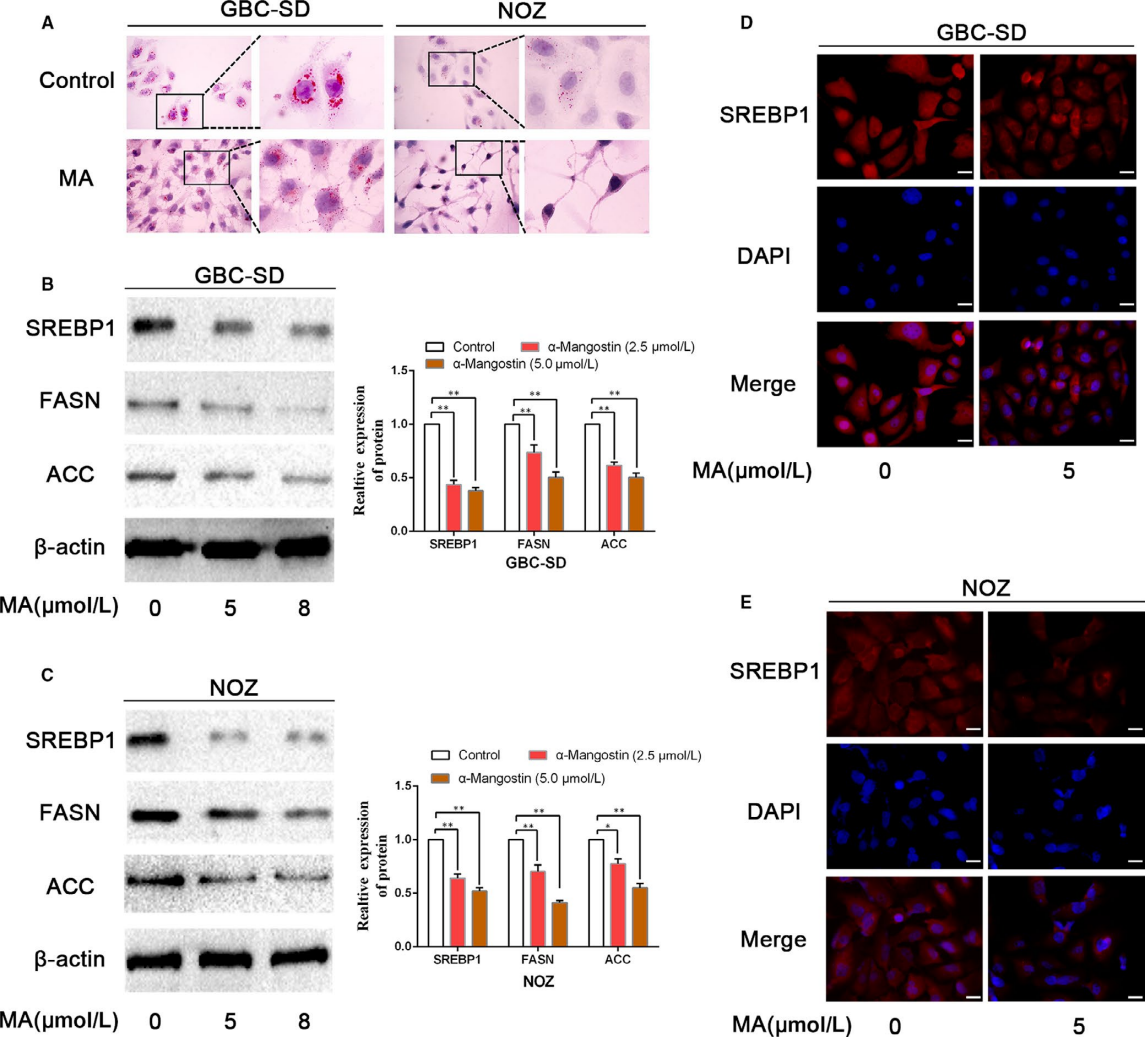
MA induced an inhibition of de novo lipogenesis in gallbladder cancer cells. A, GBC‐SD and NOZ cells were incubated with MA; the Oil Red O staining was performed to visualize the lipid droplets in cancer cells. B, C, GBC‐SD and NOZ cells were treated with MA (5 or 8 μmol/L) for 48 h; the expression levels of the key genes (SREBP1, FASN and ACC) involved in lipid metabolism were examined by WB. D, E, The effects of MA on the nuclear translocation of SREBP1 were assessed by immunofluorescence staining. Scale bar: 20 μm. *P < .05, **P < .01. MA, α‐mangostin

Increased de novo lipogenesis has been demonstrated to be crucially important in cancer cell survival and progression.^25^ Thus, we determined whether MA can efficiently inhibit lipogenesis in the gallbladder cancer cells. Oil Red O staining confirmed that MA induces an inhibition of de novo lipogenesis in the GBC cells, as the content of lipid droplets was decreased in NOZ and GBC‐SD cells treated with MA (Figure 3A). We next examined SREBP1, a key transcription factor of lipogenesis, and evaluated the expression of SREBP1 after MA treatment. A decrease in the SREBP1 expression was observed after MA treatment compared with that in the untreated cells (Figure 3B,C). Similarly, the expression levels of FASN and ACC were down‐regulated after treatment with MA. Moreover, the nuclear translocation of SREBP1 was suppressed by α‐mangostin, as revealed by immunofluorescence (Figure 3D,E).

### 3.3 α‐Mangostin induces an inhibition of lipogenesis in gallbladder cancer cells through the AMPK/SREBP1 cascades

**Figure 4:**
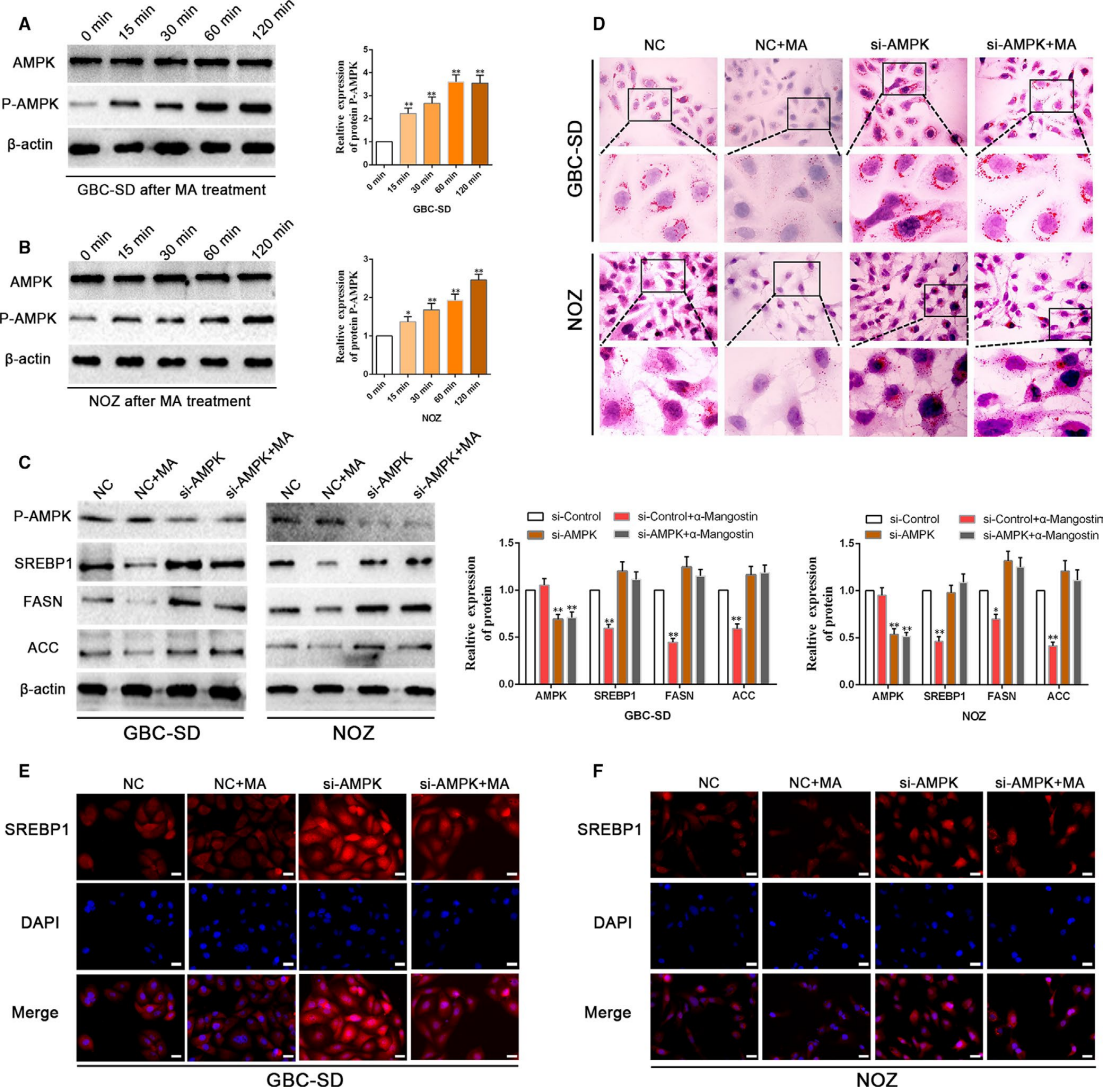
α‐Mangostin induces an inhibition of lipogenesis in gallbladder cancer cells through the AMPK/SREBP1 cascades. A, B, GBC‐SD and NOZ cells were treated with 5 μmol/L MA at various time‐points (0, 15, 30, 60 and 120 min), and the expression of total AMPK, P‐AMPK was determined by WB. C, WB analysis showed that depletion of AMPK by siRNA rescued MA‐induced repression of the expression levels of the key genes (SREBP1, FASN and ACC) involved in lipid metabolism in GBC‐SD and NOZ cells. β‐Actin was used as an internal loading control. D, The Oil Red O staining indicated that silencing of AMPK by siRNA retained MA‐induced inhibition of de novo lipogenesis in the gallbladder cancer cells. E, F, AMPK depletion increased the expression and nuclear translocation of SREBP1; inhibition of AMPK by siRNA abolished the inhibitory effects of MA on the nuclear translocation of SREBP1 in GBC‐SD and NOZ cells. Scale bar: 20 μm. *P < .05, **P < .01. MA, α‐mangostin

Previous findings have demonstrated that AMPK (AMP‐activated protein kinase) is an upstream regulator of the genes that regulate fat metabolism.^17^ AMPK activation results in inhibition of lipogenesis. Based on the existing findings, we hypothesized that the AMPK/SREBP1 cascades may mediate the effects of MA on lipogenesis in gallbladder cancer cells. To evaluate whether MA can induce the expression of p‐AMPK (phosphorylated AMPK) in GBC cells and to elucidate the time‐dependent effects of MA, we treated NOZ and GBC‐SD cells with 5 μmol/L MA. Immunoblot analysis showed that p‐AMPK expression is increased in a time‐dependent manner in GBC cells (Figure 4A,B). Thus, our data showed that MA can increase p‐AMPK expression and suppress the markers of lipogenesis. Next, we investigated whether AMPK is an integral component of the MA‐mediated inhibition of lipogenesis. We used specific siRNA to knockdown AMPK; the efficiency of silencing of AMPK is illustrated in Figure 4C. Oil Red O staining substantiated that AMPK knockdown increased the content of lipid droplets in NOZ and GBC‐SD cells and that the inhibition of lipogenesis induced by MA was reversed by AMPK knockdown (Figure 4D). Moreover, AMPK‐depleted and negative control GBC cells treated with MA were examined using immunoblot analysis. We observed that MA inhibited SREBP1, FASN and ACC expression in GBC‐SD and NOZ negative control cells (Figure 4C). AMPK knockdown cells tended to possess higher expression of SREBP1, FASN and ACC; this increase was not abrogated by MA (Figure 4C). Similarly, the immunofluorescence analysis demonstrated that the nuclear translocation of SREBP1 was repressed in GBC‐SD and NOZ negative control cells due to the effect of MA; however, MA‐induced inhibition of nuclear localization of SREBP1 was abrogated by AMPK depletion (Figure 4E,F). Together, these findings suggest that α‐mangostin induces inhibition of lipogenesis in the gallbladder cancer cells through the AMPK/SREBP1 cascades.

### 3.4 Depletion of SREBP1 potentiates gemcitabine sensitivity in GBC cells

**Figure 5:**
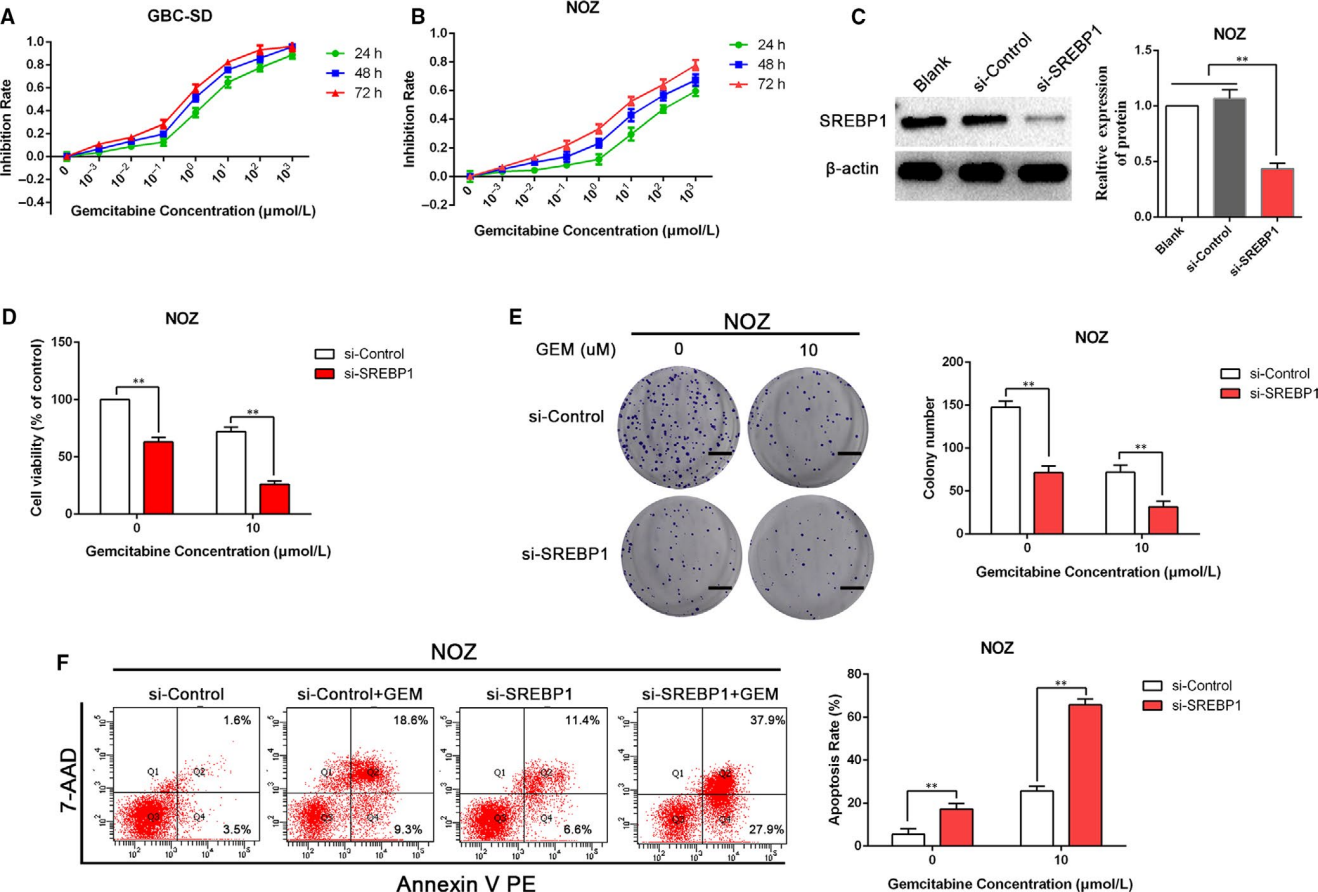
SREBP1 depletion enhances gemcitabine sensitivity in the gallbladder cancer cells. A, B, GBC‐SD and NOZ cells were incubated with a gradually increasing concentration of gemcitabine (0, 10^−3^, 10^−2^, 10^−1^, 10^0^, 10^1^, 10^2^ and 10^3^ μmol/L) for 24, 48 or 72 h; the MTT assay was used to analyse cancer cell viability. C, The efficacy of siRNAs silencing SREBP1 in NOZ cells was determined by WB analysis. D, After transfection with si‐Control or si‐SREBP1 for 48 h, cell viability was determined by the MTT assay. E, The effect of si‐Control or si‐SREBP1 combined with gemcitabine on colony formation in NOZ cells. Images are representative of three independent experiments. Scale bar: 1 cm. F, The effects of si‐SREBP1 on apoptosis of NOZ cells after treatment with 10 μmol/L gemcitabine for 48 h was examined by flow cytometry. **P < .01. GEM, gemcitabine

Owing to the evidence supporting the impact of SREBP1 on the lipogenesis, de novo lipogenesis has been validated to be essential in survival and progression of cancer cells. We next examined whether the loss of SREBP1 impacts the gemcitabine sensitivity of GBC cells. Initially, the MTT assay was performed to detect the impact of gemcitabine on the viability of GBC‐SD and NOZ cells. As shown in Figure 5A,B, GBC‐SD cells were sensitive to gemcitabine and NOZ cells were resistant to gemcitabine, which was consistent with previous findings.^4^ Thus, we utilized NOZ cells in subsequent experiments. To assess the effect of SREBP1 on gallbladder cancer cell survival and resistance to gemcitabine, we added 10 μmol/L gemcitabine to NOZ cells treated with si‐SREBP1 or si‐Control and verified the silencing of SREBP1 in NOZ cells by WB analysis (Figure 5C). The results of the MTT assay indicated that the viability of NOZ cells was lower in the si‐SREBP1 group compared with that in the si‐Control group after treatment with gemcitabine (Figure 5D). Moreover, colony formation was substantially reduced after depletion of SREBP1; SREBP1 depletion enhanced the inhibition of colony formation by gemcitabine (Figure 5E). In addition, we found that silencing SREBP1 potentiated the GEM‐induced apoptosis of NOZ cells (Figure 5F), demonstrating the importance of SREBP1 in the GEM‐mediated promotion of apoptosis in GBC cells.

### 3.5 Inhibition of SREBP1 by α‐mangostin enhances the sensitivity of GBC to gemcitabine

**Figure 6:**
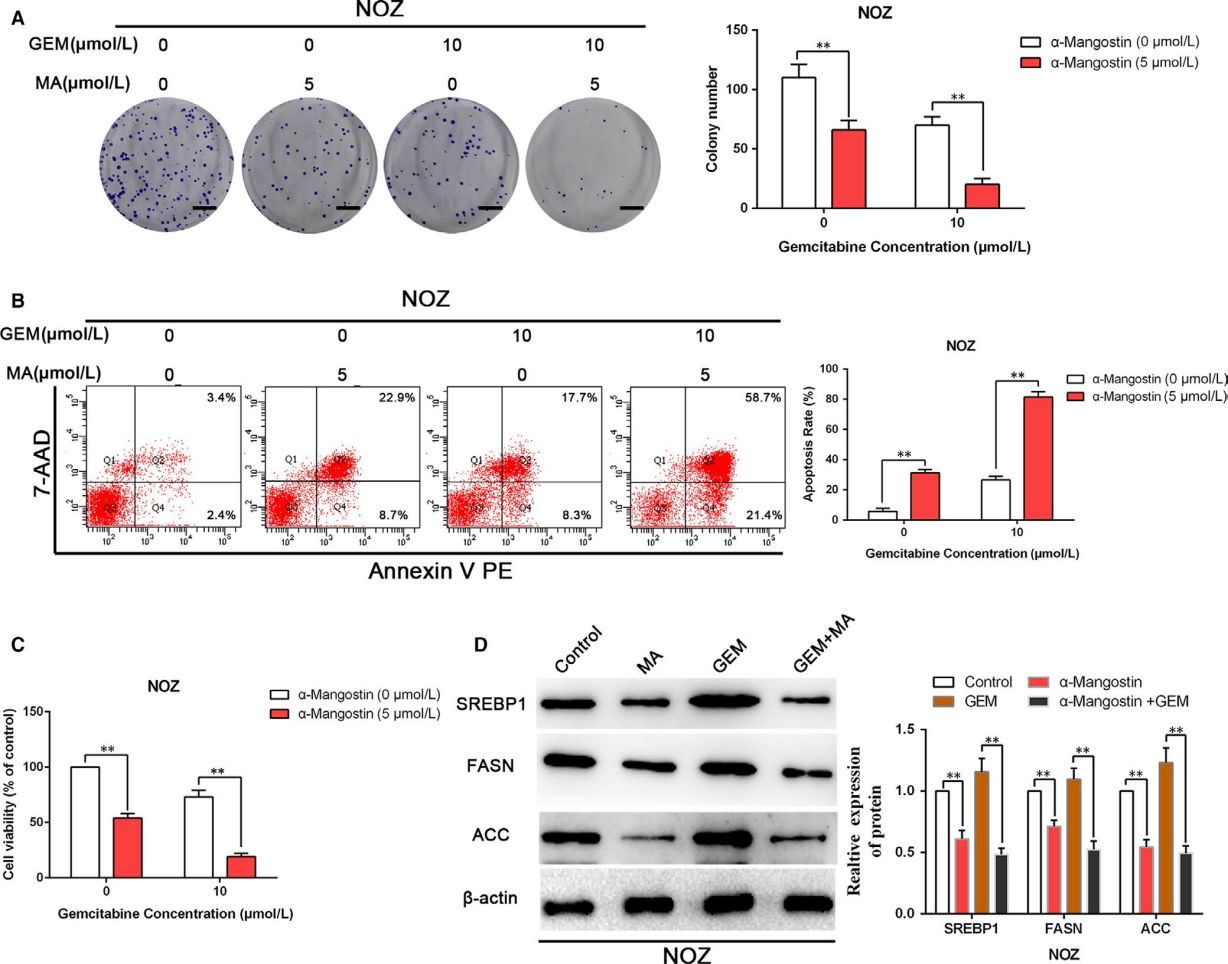
Repression of SREBP1 by α‐mangostin enhances the sensitivity of gallbladder cancer cells to gemcitabine. A, The combined effects of GEM and MA on colony formation in NOZ cells were measured. Images are representative of three independent experiments. Scale bar: 1 cm. B, The combined effects of GEM and MA on apoptosis in NOZ cells were determined by flow cytometry. C, NOZ cells were incubated with MA, GEM or MA plus GEM; then, the combined effects of MA and GEM on cell viability in NOZ cells were determined by MTT assay. D, NOZ cells were treated with GEM (10 μmol/L), MA (5 μmol/L) or GEM plus MA for 48 h; total protein was extracted to detect the expression levels of lipogenesis‐correlated genes (SREBP1, FASN and ACC). **P < .01. MA, α‐mangostin. GEM, gemcitabine

To confirm whether α‐mangostin increases the sensitivity of NOZ cells to gemcitabine, we examined colony formation of NOZ cells after treatment with MA and gemcitabine. As shown in Figure 6A, treatment with the combination of MA and gemcitabine significantly decreased the number of colonies compared with that in the case of the treatments with gemcitabine or MA alone. Similarly, treatment with MA or gemcitabine alone elicited an increase in apoptotic population of NOZ cells compared with that in the untreated control cells; almost all cells experienced apoptosis after the treatment with the combination of gemcitabine and MA (Figure 6B). Furthermore, NOZ cells were treated with 5 μmol/L α‐mangostin and 10 μmol/L gemcitabine. The results of the MTT assay indicated that the viability of cancer cells was remarkably lower in the group treated with the combination of α‐mangostin and gemcitabine than that in the groups treated with α‐mangostin or gemcitabine alone (Figure 6C). In addition, the expression levels of the genes involved in lipid metabolism, including SREBP1, FASN and ACC, in NOZ cells exposed to MA and gemcitabine were assessed by WB analysis. As shown in Figure 6D, the expression of SREBP1, FASN and ACC was substantially reduced by MA treatment; interestingly, gemcitabine treatment tended to display higher expression of SREBP1, FASN and ACC, which was consistent with previously reported results.^16^ Gemcitabine treatment enhanced the cancer stemness via elevated expression of SREBP1.^16^ However, in the group treated with the combination of gemcitabine and MA, MA can abrogate the effects of gemcitabine on the expression of SREBP1, FASN and ACC (Figure 6D). Collectively, these results illustrate that inhibition of SREBP1 activity by α‐mangostin enhances the sensitivity of GBC cells to gemcitabine.

### 3.6 Inhibition of SREBP1 induced by α‐mangostin enhances the chemosensitivity to gemcitabine in vivo

**Figure 7:**
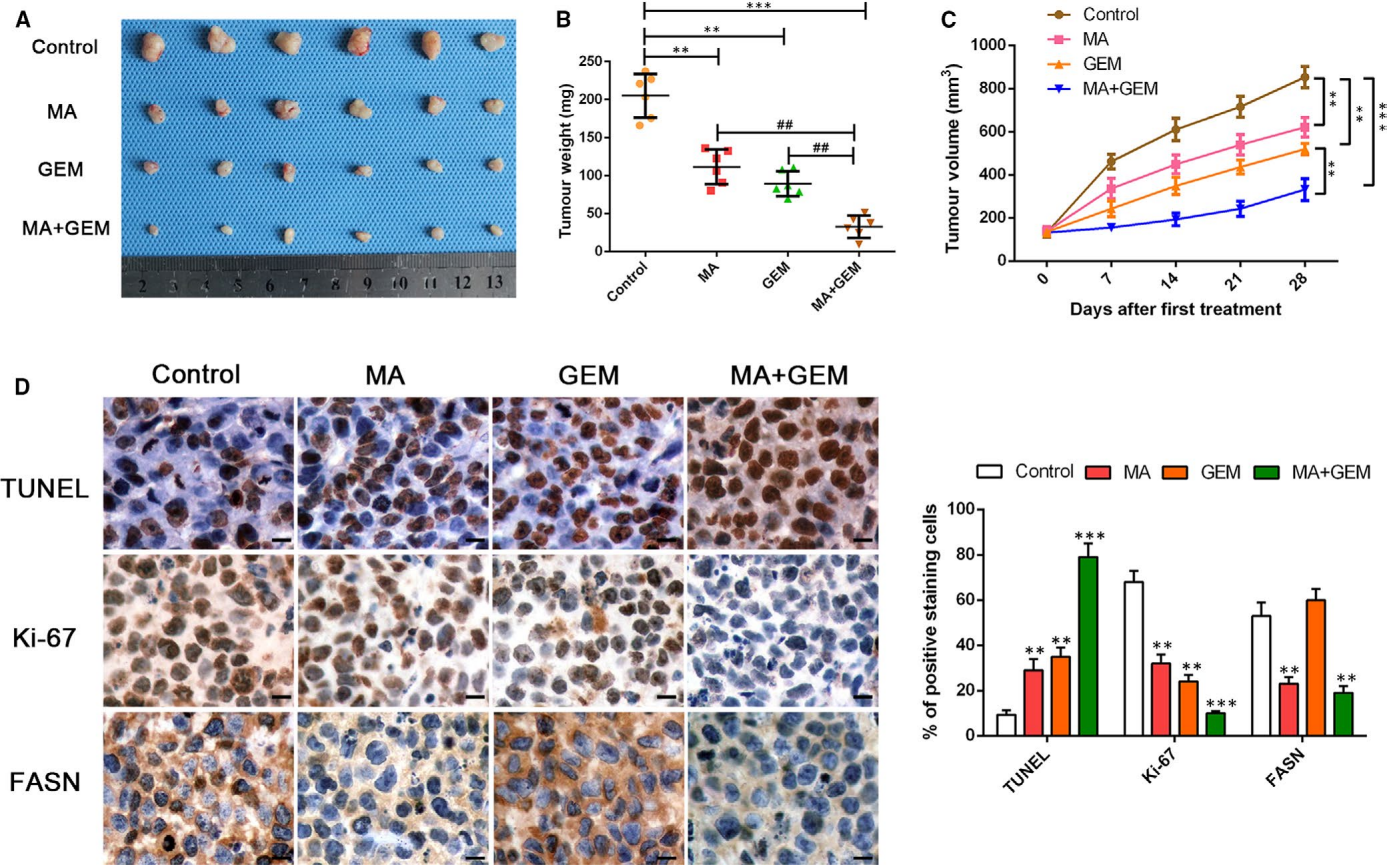
α‐Mangostin enhances the chemosensitivity of gemcitabine in vivo. A, Representative images of subcutaneous xenografts in nude mice implanted with NOZ and treated with MA, GEM and MA combined with GEM (n = 6 per group). B, C, Xenograft weight (mg) and tumour sizes were monitored and quantified. n = 6 per group, **P < .01 or ^##^ *P* < .01 by ANOVA for tumour weight; **P < .01 by repeated‐measures ANOVA for tumour sizes. D, TUNEL staining, immunohistochemistry staining and semiquantification of the data of Ki‐67 and FASN in the xenograft tissues from various groups. Magnification is ×400; the scale bar represents 20 μm. n = 6, **P < .01 or ***P < .001 by ANOVA

Our in vitro observations suggested that MA treatment may potentiate the sensitivity of GBC cells to gemcitabine in vivo. We investigated whether α‐mangostin alone or in combination with gemcitabine influences subcutaneous growth of gallbladder cancer in nude mice. Our results indicated that MA or gemcitabine administration to nude mice harbouring NOZ tumours can reduce tumour growth; moreover, MA administration can significantly potentiate gemcitabine‐induced inhibition of tumour growth, as shown in Figure 7A‐C. Tumour samples were utilized to evaluate the effect of MA or gemcitabine on proliferation. In agreement with the in vitro findings, tumours from mice treated with MA or gemcitabine alone showed decreased levels of proliferation with reduced Ki‐67 expression and elevated apoptosis confirmed by the TUNEL staining (Figure 7D). Furthermore, the inhibition of proliferation and up‐regulation of apoptosis were clearly observed in the groups treated with the combination of MA and gemcitabine. In addition, IHC of tumour samples revealed an increasing trend of FASN expression in the gemcitabine treatment group, while FASN expression was repressed in the α‐mangostin and gemcitabine combination treatment group. Collectively, these results illustrated that α‐mangostin increased the susceptibility of nude mice harbouring NOZ tumours to gemcitabine; the potential mechanism of this phenomenon may be mediated by the inhibition of the expression of the genes involved in lipogenesis.

## 4 DISCUSSION

Clinical and epidemiological findings have illustrated that obesity and being overweight are positively correlated with the high risk of gallbladder cancer (GBC),^13^, ^26^, ^27^ implying that aberrant lipid metabolism may participate in tumourigenesis and malignant progression of GBC. In fact, metabolic reprogramming in various types of cancer cells has been substantiated to confer proliferation and survival of cancer cells.^28^, ^29^ Changes in the patterns of lipid metabolism are an important metabolic rewiring phenomenon considered to be the hallmark of cancer.^29^, ^30^ Increases in lipid synthesis and uptake and of lipid storage in cancer cells can sustain rapid tumour growth.^31^ Sterol regulatory element‐binding proteins (SREBPs) are important transcription factors that regulate the expression of the genes involved in lipid synthesis and uptake; SREBPs play an important role in lipid metabolism under physiological and pathological conditions.^32^ SREBP dysregulation has been found in various types of cancer and in metabolic syndrome. Therefore, targeting a modified pathway that regulates lipid metabolism is recognized as a promising anticancer alternative.

Previous studies have verified that AMPK activation is efficient and imperative for the inhibition of SREBP‐1c cleavage, repression of the SREBP‐1c gene expression in the SRE‐dependent mode, suppression of nuclear migration and suppression of the gene expression of FASN.^17^ It should be mentioned that AMPK activation causes SREBP‐1c Ser372 phosphorylation to impair SREBP‐1c cleavage and inhibits SREBP‐1c gene expression.^17^ Therefore, we propose that the activation of AMPK is a potential therapeutic strategy in cancer; identification of efficient agents targeting the AMPK/SREBPs signalling pathway may be a useful strategy to improve the treatment outcome of patients suffering from GBC. Intriguingly, our results indicate that α‐mangostin repressed proliferation and colony formation, induced cell cycle arrest and apoptosis and suppressed de novo lipogenesis in the GBC cells. Additionally, the NOZ and GBC‐SD cells were treated with MA increased the p‐AMPK expression in a time‐dependent manner; AMPK depletion can reverse the MA‐induced inhibition of the expression of SREBP1, FASN and ACC, indicating that MA, as a dietary xanthone, is sufficient for stimulation of AMPK phosphorylation and subsequent inhibition of SREBP activity.

Gallbladder cancer is a relatively rare but highly lethal neoplasm, lacking effective strategies to conquer this disease. Patients suffering from GBC usually have a few signs or symptoms; however, their condition can deteriorate rapidly due to the development of metastasis.^33^ Currently, gemcitabine/cisplatin has been recognized as the preferred regimen for the first‐line treatment of patients suffering from advanced biliary tract cancers, including gallbladder cancer.^3^ However, patients undergoing the first‐line chemotherapy often have a rapidly worsening performance status; only a small number of patients will be suitable for subsequent treatment.^3^ Here, we show that α‐mangostin is effective in inhibiting lipogenesis and increasing sensitivity to gemcitabine via SREBP1 suppression in gallbladder cancer cells in vitro; then, we evaluated how α‐mangostin alone or in combination with gemcitabine influences the subcutaneous GBC growth in nude mice. Our results demonstrated that MA or gemcitabine administration to nude mice harbouring NOZ tumours can reduce tumour growth; moreover, MA administration can significantly potentiate gemcitabine‐induced inhibition of tumour growth. Hence, α‐mangostin appears to be a good candidate for further development as an effective anticancer drug for the therapeutic treatment of GBC. Nevertheless, the detailed molecular mechanisms of the antitumour effects of α‐mangostin and its therapeutic safety in humans require further studies. Furthermore, our findings can potentially open new avenues of research on the role of α‐mangostin as a novel inhibitor of lipogenesis in gallbladder cancer.

## CONFLICT OF INTEREST

No conflicts of interest exist.

## AUTHOR CONTRIBUTIONS

EL and DD conceived and designed the experiments; YS, YF, YH, JJ and CW performed the experiments; YW and XD analysed the data; QG contributed reagents/materials/analysis tools; YS, EL and DD wrote the paper. All authors read and approved the final manuscript.

## Data Availability

The data that support the findings of this study are available from the corresponding author upon reasonable request.
